# Real-World Multicenter Experience in Tumor Debulking Prior to Blinatumomab Administration in Adult Patients With Relapsed/Refractory B-Cell Precursor Acute Lymphoblastic Leukemia

**DOI:** 10.3389/fonc.2021.804714

**Published:** 2022-01-06

**Authors:** Massimiliano Bonifacio, Cristina Papayannidis, Federico Lussana, Nicola Fracchiolla, Mario Annunziata, Simona Sica, Mario Delia, Robin Foà, Giovanni Pizzolo, Sabina Chiaretti

**Affiliations:** ^1^ Department of Medicine, Section of Hematology, University of Verona, Verona, Italy; ^2^ “Serágnoli” Institute of Hematology, Istituto di Ricovero e Cura a Carattere Scientifico (IRCCS), Azienda Ospedaliero-Universitaria di Bologna, Bologna, Italy; ^3^ Hematology and Bone Marrow Transplant Unit, Azienda Socio-Sanitaria Territoriale Papa Giovanni XXIII, Bergamo, Italy; ^4^ Oncohematology Unit, Fondazione Istituto di Ricovero e Cura a Carattere Scientifico (IRCCS) Ca’ Granda Ospedale Maggiore Policlinico, University of Milan, Milano, Italy; ^5^ Hematology Division, Azienda Ospedaliera di Rilievo Nazionale Cardarelli, Naples, Italy; ^6^ Department of Diagnostic Imaging, Oncological Radiotherapy and Hematology, Fondazione Policlinico A. Gemelli Istituto di Ricovero e Cura a Carattere Scientifico (IRCCS), Rome, Italy; ^7^ Department of Radiological and Hematological Sciences, Hematology Unit, Università Cattolica del Sacro Cuore, Rome, Italy; ^8^ Hematology and Stem Cell Transplantation Unit, Azienda Ospedaliero-Universitaria Consorziale Policlinico, Bari, Italy; ^9^ Hematology, Department of Translational and Precision Medicine, Sapienza University of Rome, Rome, Italy

**Keywords:** B-cell precursor acute lymphoblastic leukemia, relapsed/refractory, debulking, blinatumomab, real-world, chemotherapy

## Abstract

Blinatumomab is an immunotherapeutic agent with dual specificity for CD3 and CD19 that is approved for the treatment of relapsed/refractory B-cell precursor acute lymphoblastic leukemia (R/R B-ALL). A steroid based pre-treatment is recommended before administering blinatumomab to patients with a high tumor burden to minimize the risk of tumor lysis syndrome, but the optimal debulking regimen and whether it can improve responses remain unclear. The present study retrospectively evaluated real-world outcomes following tumor debulking and blinatumomab infusion in R/R B-ALL adult patients treated at 7 Italian centers. Data were collected from 34 patients. The choice of the cytoreductive therapy was made by the treating clinician on an individual patient basis; regimens included chemotherapy (n=23), steroids (n=7) and tyrosine kinase inhibitors alone or in combination (n=4). The rate of complete responses (CR) and complete minimal residual disease (MRD) responses in CR patients were 67.6% and 81% respectively, after 2 cycles of blinatumomab. Moreover, among patients with a high tumor burden 50% obtained a CR, with 89% of them also achieving a complete MRD response. Favorable responses were also obtained in patients over 50 years of age at treatment initiation. Overall, 7 of 23 patients in CR after blinatumomab underwent hematopoietic stem cell transplantation. The results of this retrospective study highlight the heterogeneity in the use of pre-blinatumomab tumor debulking in real-life clinical practice. Nonetheless, debulking pre-treatment enhanced responses to blinatumomab compared to historic studies, indicating that this strategy may help to improve outcomes for R/R B-ALL patients.

## 1 Introduction

B-cell precursor (BCP) acute lymphoblastic leukemia (B-ALL) accounts for almost 75% of all cases of adult ALL (1). Despite significant improvements in the management of B-ALL patients in the last decades, the long-term survival for adults is still around 50% (2). In fact, even though the proportion of adult patients achieving a first complete remission with frontline treatment is 80-90%, about half of them subsequently relapse (3). Relapsed or refractory B-ALL (R/R B-ALL) is associated with poor event-free survival (EFS), overall survival (OS) and a cure rate of less than 10% (4–6), and its treatment thus remains a clinical challenge (3). This is attributable to the persistence of chemoresistant minimal residual disease (MRD) following initial chemotherapy, which increases the risk of hematologic relapse and is regarded as the most important prognostic factor in ALL (5, 7–9).

Novel agents for the treatment of B-ALL include blinatumomab, a bispecific T-cell engager with a dual specificity for CD19 and CD3 that directs cytotoxic T cells activity against malignant CD19-positive B cells. Blinatumomab was first approved by the Food and Drug Administration (FDA) for the treatment of R/R B-ALL and later for B-ALL patients with MRD (10).

The efficacy of blinatumomab in adults with R/R B-ALL has been demonstrated in the pivotal phase III TOWER study that randomized 405 patients to either blinatumomab monotherapy or standard of care chemotherapy (11). The rate of complete hematologic responses (CHR) and the overall response rates were higher in the blinatumomab arm than in the comparator arm (34% vs. 16% and 44% vs. 25% respectively). Among responders, MRD negativity rates were 76% for blinatumomab and 48% for chemotherapy, while median OS was 7.7 months for blinatumomab treated patients vs. 4 months for the chemotherapy group. Blinatumomab also demonstrated efficacy for the treatment of adults with Philadelphia-positive (Ph+ ALL) R/R B-ALL patients. In the phase 2 single-arm multicenter study ALCANTARA, 35.6% patients obtained a complete remission (CR) with partial hematological recovery with a rate of complete MRD responses of 87.5% (12). After a median follow-up of 16.1 months, the median relapse-free survival (RFS) was 6.8 months, while the median OS was 9.0 months in the entire cohort and 19.8 months in blinatumomab responders (13).

In patients with R/R disease, a high tumor burden is associated with a lower response rate and an increased risk of cytokine release syndrome (CRS) (14). Before initiation of blinatumomab, it is recommended to administer a steroid pre-treatment to patients with bone marrow blasts (BMB) above 50% in order to reduce the load of disease and prevent treatment-related adverse reactions (15). However, whether a pre-treatment phase can lead to higher responses to blinatumomab and the optimal debulking regimen to use still remain unclear. The purpose of the present study was to evaluate the response to blinatumomab following prior debulking therapy in R/R B-ALL adult patients treated in the clinical practice in Italy. These results will likely represent the first step towards the standardization of the use of a debulking pre-phase for patients with aggressive disease treated with blinatumomab.

## 2 Materials and Methods

### 2.1 Study Design

In order to collect real-world data on the use of debulking prior to blinatumomab, an *ad hoc* survey was developed and shared among 7 Italian centers with extensive expertise in the treatment of ALL. All patients were ≥18 years at diagnosis and with R/R B-ALL. The survey collected data from previously approved observational studies on patients treated with blinatumomab from January 2014 to February 2020 and included disease characteristics at treatment initiation, the cytoreductive regimen administered, and responses to blinatumomab. All patients enrolled in those studies at the participating centers were screened and all patients who performed a debulking treatment prior to blinatumomab administration were selected for the present study. A total of 34 patients were included in this analysis. Of these, 14 were treated in the expanded access NEUF study (16), 15 in the Italian post-approval access program (PAAP), and 5 initiated treatment in routine clinical practice as part of an observational study on the real-world use of blinatumomab (17). All patients provided a written informed consent for participation in the studies or the PAAP. Each cycle consisted of 4 weeks of continuous infusion of blinatumomab followed by 2 weeks of wash-out. Patients received 9 mcg/day for the first 7 days of the first cycle and 28 mcg/day afterwards. The choice of administering debulking treatment, as well as the type of cytoreductive regimen, were left to the investigator’s choice. Patients were grouped according to the following criteria: a) those who received high-dose steroids only; b) those who received non-intensive chemotherapy (non-myelotoxic or moderately myelotoxic agents) regimens with or without steroids; c) those who received intensified chemotherapy regimens (polychemotherapy, generally associated with significant myelotoxic action); d) Ph+ ALL patients who received a tyrosine kinase inhibitor (TKI) alone, with steroids, or with chemotherapy.

Patients were evaluated after 2 cycles of blinatumomab for hematological responses and MRD responses on bone marrow aspirates. According to the criteria of TOWER study (11), CR was defined as 5% or less BMB and no evidence of extramedullary disease, and was further characterized according to the extent of recovery of peripheral blood counts as follows: CR with full recovery (platelets >100,000/μL and absolute neutrophil count >1,000/μL), CR with partial recovery (CRp: platelets >50,000/μL and absolute neutrophil count >500/μL), or CR with incomplete recovery (CRi: platelets >100,000/μL or absolute neutrophil count >1,000/μL). MRD analysis was carried out locally through allele-specific real-time quantitative polymerase chain reaction (PCR) using patient-specific molecular probe(s) and/or multiparameter flow cytometry (FCM). The sensitivity of MRD assays was ≥10^-4^ (18). Complete MRD response was defined as the absence of target transcript amplification by PCR and/or absence of detectable leukemic cells by FCM. High tumor burden before blinatumomab was defined as BMB ≥50%.

## 3 Results

### 3.1 Patient Characteristics

Baseline characteristics are summarized in **Table 1**. Median age at treatment initiation was 45 years (range 20-75); 13 patients had Ph+ ALL (38.2%); 70.6% of patients had relapsed and 29.4% had refractory B-ALL disease. The rate of prior hematopoietic stem cell transplantation (HSCT) was 50%. The median number of previous treatment lines was 2 (range 1-5) and 79% of patients had received 2 or more prior therapies. The median BMB at baseline was 69% (range 6%-90%), 58.8% of patients had BMB ≥50%.

**Table 1:** Patient characteristics.

	N=34 n (%)
**Sex**	
Male	21 (61.8%)
Female	13 (38.2%)
**Age**	
<50	19 (55.9%)
≥50	15 (44.1%)
**Disease status**	
Relapsed	24 (70.6%)
Refractory	10 (29.4%)
**Previous treatment lines**	
1	7 (20.6%)
2	13 (38.2%)
≥3	14 (41.2%)
**Prior HSCT**	
Yes	17 (50%)
No	17 (50%)
**Bone marrow blasts**	
<20%	7 (20.6%)
20-49%	6 (17.7%)
≥50%	20 (58.8%)
Not available	1 (2.9%)
**Ph status**	
Positive	13 (38.2%)
Negative	21 (61.8%)

HSC, Hematopoietic stem cell transplantation.

### 3.2 Debulking Treatments

The different debulking regimens are summarized in **Table 2**. Overall, 20.6% patients received steroids alone and 52.9% patients (18/34) received a pre-treatment regimen with low-dose chemotherapy with or without steroids; four of the 13 Ph+ ALL patients received a TKI (1 with steroids, 1 without steroids, and 2 in combination with chemotherapy). In patients treated with polychemotherapy the median time from the start of the debulking regimen and the blinatumomab administration was 21 days (range 20-48). Patients with a high tumor load mostly received low intensity chemotherapy with or without steroids (14/20; 70%).

**Table 2:** Debulking therapies administered.

Debulking therapies	Patients	
	N	Total, n/N (%)
Steroids		
- Dexamethasone	5	7/34 (20.6%)
- Prednisone	2	
Low-intensity chemotherapy with or without steroids		
- Vincristine + steroids	6	18/34 (52.9%)
- Vindesine + dexamethasone	1	
- 6-MP + steroids	2	
- Vincristine	4	
- HU	1	
- Vinblastine + prednisone + 6-MP + methotrexate	1	
- Vincristine + 6-MP	2	
- steroids + HU + 6-MP	1	
Polychemotherapy		
- Idarubicin + vincristine + PEG-asparaginase + dexamethasone	1	5/34 (14.7%)
- Hyper-CVAD regimen	1	
- Vincristine + daunoblastin + erwinase + methylprednisolone	1	
- Cyclophosphamide + vincristine	1	
- Steroids + cyclophosphamide + vincristine	1	
TKI with or without steroids or with chemotherapy		
- Ponatinib	1	4/34 (11.8%)
- Ponatinib + dexamethasone	1	
- Vincristine + ponatinib + prednisone	1	
- Steroids + FLA-Ida + ponatinib	1	

6-MP, 6-mercaptopurine; hydroxyurea, HU; hyper-CVAD, cyclophosphamide, vincristine sulfate, doxorubicin (adriamycin) and dexamethasone; FLA-Ida, fludarabine-idarubicin; TKI, tyrosine kinase inhibitor.

Data on tumor burden after debulking and prior to the administration of blinatumomab were available for 22 of 34 patients. 18/22 patients (81.8%) had less than 20% BMB, 2 (9.1%) had 20-49% BMB, and 2 (9.1%) had ≥50% BMB. The median BMB for these 22 patients after debulking was 8% (range 0-80%).

### 3.3 Blinatumomab Treatment and Effectiveness

#### 3.3.1 Overall Responses

Of the 34 patients, 30 (88%) received blinatumomab at the full recommended dose without prolonged (i.e. >72 hours) treatment interruptions, 3 (8.8%) received the full dose with prolonged treatment interruptions due to adverse events, and 1 (2.9%) received a reduced dose. Twenty-five patients received 2 cycles of blinatumomab. The remaining 9 cases progressed during or immediately after the first cycle and did not receive the second cycle.

Twenty-three of the 34 patients (67.6%) achieved a CR within 2 cycles of blinatumomab, which was further classifiable as CR with full recovery in 19 patients (55.9%), CRp in 2 patients (5.9%) and CRi in 2 patients (5.9%). Among the 21 patients available for MRD assessment, the rate of complete MRD response was 81% (17/21).

The incidence of clinically significant CRS, mostly represented as occurrence of fever higher than 39°C, coupled or not with hypotension, was 14.7% (5 cases). Only one patient experienced a grade 3 CRS according to the CTCAE v.5.0 criteria. There were no differences in the debulking treatments among cases experiencing CRS vs those not experiencing it: indeed, the rate of patients receiving non-intensive treatments was 60% and 53.5%, high-dose steroids only was 0% and 21.4%, polychemotherapy was 20% and 14.2% and TKI was 20% and 10.7%, respectively. As for BMB percentage, all but one case of patients with CRS had ≥50% blasts before debulking and <20% blasts after debulking and prior to blinatumomab.

#### 3.3.2 Responses to Blinatumomab According to the Debulking Treatment

Among the debulking regimens, the highest rates of CR after blinatumomab were observed in patients receiving polychemotherapy (5/5; 100%) and low-intensity chemotherapy with or without steroids (11/18; 61%), and a lower percentage of responses was observed in those receiving steroids alone (3/7; 42.9%); nevertheless, this difference did not translate into a different rate of MRD responses (4/5, 7/9 and 3/3 patients, respectively). After achievement of CR with blinatumomab, 7/23 patients underwent a HSCT; among the remaining 16 patients, 8 did not undergo HSCT and 8 had received a previous transplant (HSCT was planned for 1 patient, but no donor was available).

#### 3.3.3 Responses According to Bone Marrow Blasts at Baseline and Following Debulking

**Figure 1:**
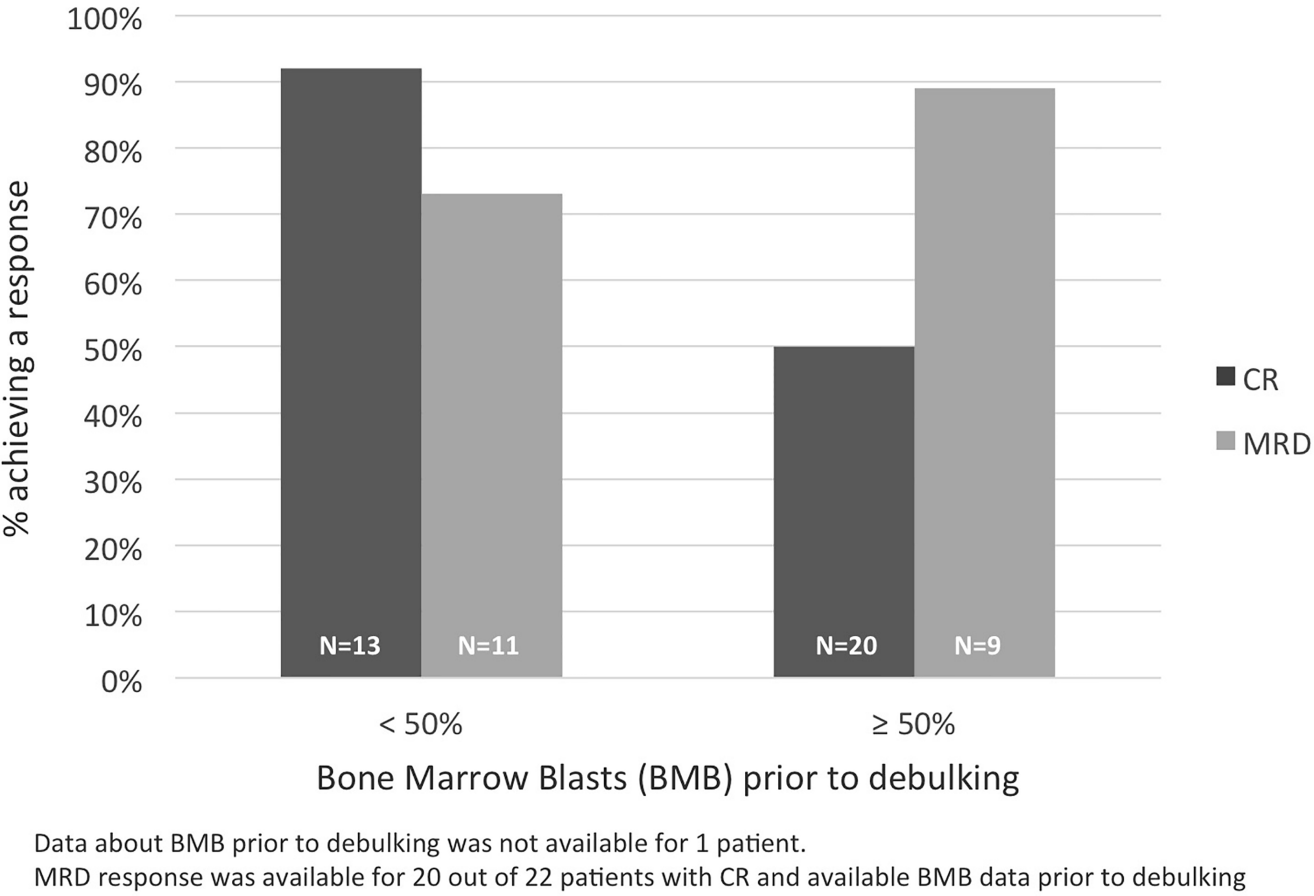
CR and MRD responses according to baseline bone marrow blasts.

CR and MRD responses were also analyzed according to the baseline BMB. Ninety-two % (12/13) of patients with pre-debulking BMB <50% reached a CR, while the rate of hematologic responses in patients with a bone marrow involvement ≥50% at baseline was 50% (10/20; p=0.02). The rates of complete MRD response in CR responders was similar among the two groups (73% and 89% of evaluable patients with pre-debulking BMB <50% and ≥50%, respectively) (**Figure 1**).

Considering the 22 patients with available BMB data after debulking and prior to blinatumomab administration, 14 out of 18 patients (78%) with <20% BMB, as well as the 2 patients with BMB 20-49% achieved a CR; contrariwise, none of the 2 patients with ≥50% BMB achieved a CR (p=0.03). A complete MRD response was obtained in 83% (10/12) of cases with BMB <20% and in both cases with BMB 20-49%.

#### 3.3.4 Response According to Patient Age

Among patients <50 years, 13 of 19 patients (68.4%) reached a CR within 2 cycles of blinatumomab and of the 11 patients evaluated for molecular response, 10 (90.9%) achieved a MRD negativity. Likewise, among patients ≥50 years, 66.7% (n=10/15) reached a CR within 2 cycles of blinatumomab, associated to a MRD negativity in 7 (70%).

#### 3.3.5 Response According to Previous Lines of Treatment

The number of lines of treatment received before debulking and blinatumomab influenced the likelihood of CR achievement. Indeed, 17 out of 20 (85%) who had received ≤2 previous lines of treatment obtained a CR, while only 6 out of 14 (42.9%) patients with >2 previous lines of treatment achieved it (p=0.02). Instead, the rate of complete MRD response among CR did not differ between the two groups (81.3% and 80% of evaluable patients with ≤2 and >2 previous lines of treatment, respectively).

## 4 Discussion

Blinatumomab has a well-documented efficacy in the treatment of Ph- and Ph+ R/R B-ALL patients (11, 13). A steroid-based pre-phase is indicated for patients with high leukemic blasts in order to minimize the risk of tumor lysis syndrome and CRS (15). Nonetheless, whether tumor debulking prior to blinatumomab can improve responses to treatment, and if so the optimal regimen still remains unclear. In an attempt to address these questions, in the present paper data on 34 adult patients with R/R B-ALL who underwent prior debulking therapy and then received blinatumomab outside of clinical trials from 7 hematology centers in Italy were collected and analyzed. Despite differences in the debulking regimen, cytoreductive therapy prior to blinatumomab treatment appeared to be an effective and safe approach to reduce tumor burden in patients with high BMB. However, it is worth noting that data on BMB before administration of blinatumomab were not available in about one-third of patients, as this additional assessment was not routinely performed.

Importantly, we observed a lower response rate to blinatumomab in patients who received steroids alone compared to those receiving chemotherapy. A steroid-based tumor debulking is recommended for patients with a high tumor load, but it is not clear if this can also improve responses to blinatumomab. In a previous pivotal study on the efficacy and safety of blinatumomab, patients with a high tumor burden received dexamethasone 10 mg/m^2^ for up to 5 days in order to reduce treatment-related adverse events (19). The authors reported no correlation between steroid administration and later responses to treatment, even though the extent of blasts reduction after debulking was not measured (19). In a real-world chart review of 14 patients, cytoreductive therapy (dexamethasone alone or in combination with chemotherapy) given before blinatumomab to patients with ≥50% BMB appeared safe, but it was not possible to determine its impact on outcomes due to the small number of patients enrolled (20). On the other hand, in a small study on 9 pediatric patients with B-ALL who relapsed after HSCT, the 2 patients who received debulking chemotherapy after lack of response to the first blinatumomab cycle drastically reduced their tumor burden and achieved a complete MRD response after a subsequent cycle of blinatumomab, which allowed for a successful bridging to a second HSCT (21). This finding supports the hypothesis that a chemotherapy-based tumor debulking regimen can improve responses to subsequent immunotherapy. As chemotherapy agents have been shown to deplete the number of regulatory T cells (22–24), which in turn hamper blinatumomab-mediated proliferation of cytotoxic T cells (25), the administration of such debulking regimen ahead of blinatumomab may further enhance the effectiveness of immunotherapy. At variance, the role of TKIs appears to be different *in vitro* (26) and *in vivo* (27, 28), as *in vivo* dasatinib treatment appeared to upmodulate cytotoxic activity, contributing to the high rates of molecular response observed in Ph+ ALL patients treated frontline with dasatinib and blinatumomab without the use of systemic chemotherapy (27, 28).

Overall, we observed better hematologic responses to blinatumomab in patients who received debulking with polychemotherapy, which suggests that polychemotherapy does not hamper the T-cell function even after regimens using multiple drugs (28), even though a formal evaluation of T- and B-cell kinetics was not carried out. However, in this series the use of a debulking regimen with low-intensity chemotherapy (with or without steroids) allowed to achieve relatively high CR rates and comparable MRD negativity rates without delaying the use of blinatumomab, while reducing the risk of adverse events related to myelosuppressive chemotherapy; thus, low-intensity chemotherapy might represent a preferable strategy to balance effectiveness and risks.

In this study, the high rates of hematological responses and complete MRD responses observed after a debulking pre-treatment followed by 2 cycles of blinatumomab compare favorably with historical studies of blinatumomab in adult patients with R/R B-ALL, in which the rates of CR ranged between 36% and 45% (11, 12, 19, 29). Patients with a poor prognosis, such as those with a high baseline tumor load and the elderly, also responded well to blinatumomab; in fact, 50% of patients with baseline BMB ≥50% achieved a CR, which is higher than the rate of 33% reported for this patient subgroup in a recent meta-analysis on the efficacy of blinatumomab (29), reinforcing the notion that lowering the blast count before blinatumomab leads to a higher efficacy of the drug (10, 11). Notably, 89% of such patients achieved a MRD negativity, which confirms that blinatumomab is capable of inducing deep responses even in patients with a high tumor burden. In our analysis, responses to blinatumomab in elderly patients were in line with the overall study population.

We could also confirm that the load of previous treatment has an impact. Indeed, patients with ≤2 lines of previous therapy appeared to have better responses than those with >2 lines of prior treatment, as already observed in a sub-analysis of the TOWER study (30).

The present survey has two main limitations: the small number of patients analyzed and the retrospective nature of the study. Moreover, the impact of debulking on long-term outcomes, such as relapse-free survival and OS, needs to be further evaluated. In light of the favorable response rates reported herein, future investigations will be aimed at determining the optimal debulking regimen prior to blinatumomab treatment, as this could significantly improve the outcomes of patients for whom therapeutic options are very limited.

In conclusion, we report that a debulking pre-phase can improve responses to blinatumomab in adult patients with R/R B-ALL, with a particularly pronounced benefit for patients with a high disease burden and for the elderly. Our findings also indicate that chemotherapy-based therapies, either of standard or reduced intensity with or without steroids, are more effective than those employing steroids only, thus suggesting that minimally or moderately myelotoxic agents might be preferable to achieve effective tumor debulking while reducing the risks of adverse events, which in turn could delay the use of blinatumomab. Nonetheless, further investigations are needed to confirm the optimal debulking regimen for patients receiving blinatumomab infusion.

## Data Availability Statement

The raw data supporting the conclusions of this article will be made available by the authors, without undue reservation.

## Ethics Statement

The studies involving human participants were reviewed and approved by Comitato Etico per la Sperimentazione Clinica delle Province di Verona e Rovigo - ref. 1710CESC. The patients/participants provided their written informed consent to participate in this study.

## Author Contributions

Conceptualization and design: MB, GP, and SC. Data acquisition: MB, CP, FL, NF, MA, SS, MD, and SC. Data analysis and interpretation: MB, RF, GP, and SC. Writing (original draft): MB, GP, and SC. Writing (review and editing): all. All authors contributed to the article and approved the submitted version.

## Funding

This work was partly supported by Progetti di Rilevante Interesse Nazionale (PRIN) Italia, 2017PPS2X4 project (MB and SC) and by Associazione Italiana Ricerca sul Cancro (AIRC), Special 5x1000 Program Metastases (21198), Milan, Italy (RF).

## Conflict of Interest

MB declares honoraria (participation in advisory boards) from Amgen, Bristol Myers Squibb, Novartis, and Pfizer. CP declares honoraria (participation in advisory boards) from Abbvie, Amgen, Novartis, Pfizer and honoraria from Amgen, Astellas, Novartis, and Pfizer. FL declares personal fees (in advisory boards or sponsored lectures) from Amgen, Astellas, Janssen, Jazz Pharmaceutical, and Pfizer. NF declares honoraria (speakers bureau) from Amgen, Gilead, Jazz Pharmaceutical, and Pfizer. SS declares honoraria from Alexion, Amgen, and Jazz Pharmaceuticals; MD declares personal fees (sponsored lectures) from Amgen. RF declares honoraria (advisory boards speakers bureau) from Amgen, Astrazeneca, Novartis, Incyte, Janssen, and Pfizer. GP declares honoraria from AbbVie, Amgen, Astrazeneca, Janssen-Cilag, Celgene, and Incyte. SC declares honoraria (in advisory boards) from Abbvie, Amgen, Incyte, Novartis, and Pfizer.

The remaining author declares that the research was conducted in the absence of any commercial or financial relationships that could be construed as a potential conflict of interest.

## Publisher’s Note

All claims expressed in this article are solely those of the authors and do not necessarily represent those of their affiliated organizations, or those of the publisher, the editors and the reviewers. Any product that may be evaluated in this article, or claim that may be made by its manufacturer, is not guaranteed or endorsed by the publisher.
